# Coating and Density Distribution Analysis of Commercial Ciprofloxacin Hydrochloride Monohydrate Tablets by Terahertz Pulsed Spectroscopy and Imaging

**DOI:** 10.1007/s12247-012-9130-1

**Published:** 2012-05-25

**Authors:** Tomoaki Sakamoto, Alessia Portieri, Donald D. Arnone, Philip F. Taday, Toru Kawanishi, Yukio Hiyama

**Affiliations:** 1 Division of Drugs, National Institute of Health Sciences, Tokyo, 158-8501 Japan; 2 TeraView Ltd., Cambridge, CB4 0WS UK

**Keywords:** Terahertz pulsed spectroscopy, Terahertz pulsed imaging, Coating, Density distribution, Tablet, Imaging methods, Ciprofloxacin

## Abstract

Terahertz pulsed spectroscopy was used to qualitatively detect ciprofloxacin hydrochloride monohydrate (CPFX·HCl·H_2_O) in tablets, and terahertz pulsed imaging (TPI) was used to scrutinize not only the coating state but also the density distribution of tablets produced by several manufacturers. TPI was also used to evaluate distinguishability among these tablets. The same waveform, which is a unique terahertz absorption spectrum derived from pure CPFX·HCl·H_2_O, was observed in all of the crushed tablets and in pure CPFX·HCl·H_2_O. TPI can provide information about the physical states of coated tablets. Information about the uniformity of parameters such as a coating thickness and density can be obtained. In this study, the authors investigated the coating thickness distributions of film-coated CPFX·HCl·H_2_O from four different manufacturers. Unique terahertz images of the density distributions in these commercial tablets were obtained. Moreover, B-scan (depth) images show the status of the coating layer in each tablet and the density map inside the tablets. These features would reflect differences resulting from different tablet-manufacturing processes.

## Introduction

The electro-magnetic wave on terahertz region is generally defined from 0.1 THz to 10 THz (3.3 to 333 cm^−1^). This electro-magnetic region has also been known as a far-infrared wave region. But, an irradiated light energy from a typical far-infrared spectrometer equipped with a high-pressure mercury lamp will drop at a frequency below 1 THz drastically. Recent development of laser devices and semi-conductors has allowed us to use coherent terahertz wave with lower frequency. In a terahertz region, vibrational information about weak intermolecular energy such as crystal lattice, hydrogen bonding, and van der Waals force can be detected [1–6]. This leads to applications in the pharmaceutical and chemical industries such as the detection of polymorphs [2, 7–13]. A number of authors have shown that unique terahertz spectra can be obtained for active pharmaceutical ingredients (APIs), illegal drugs, and explosives [7, 9, 12]. The assignment of spectroscopic bands in this region of the spectrum remain challenging due to the complicated properties of crystalline materials, but a number of groups are having some success. Comparative studies between hydrates and their anhydrides have been reported by Kogermann et al. [14] and others [15, 16]. These authors have also investigated the thermodynamics of phase transformation following dehydration.

A time domain terahertz technology (terahertz pulsed technology) is non-destructive analytical tool for investigating pharmaceutical materials and products. This technique can provide two modes which are an imaging mode known as terahertz pulsed imaging (TPI) and a spectroscopic mode known as terahertz pulsed spectroscopy (TPS). Especially, TPI can produce images or maps which are obtained by detecting reflected pulses from each pixel on a tablet or other dosage forms. Terahertz pulses are irradiated at each pixel on a tablet and penetrate, and echoes or reflections from layers are measured. Then, TPI also obtain depth information at each pixel. The detection time and intensity of reflected wave is affected by the refractive index of the sample. For a coated tablet, this time-of-flight technique makes detector distinguish different arriving time of terahertz pulse. The reflected pulses which are originated from the interface between coating layer and the surface of core tablet or another coating layer in the tablet are detected, and information of the time of flight is used not only to calculate the coating thickness but also to acquire 3D images of a coated tablet. Ho et al. [17–19] reported that not only the coating thickness but also the density of the coating can influence the quality performance of sustained-release film-coated tablets. The authors were able to use the intensity of the terahertz reflected pulse from a coating to model the changes in refraction of terahertz pulsed wave which is correlated with changes in density of coating [19–21]. Recently, we applied TPI to the nondestructive testing of a transdermal drug delivery system. These products have a crystal reservoir system inside a membrane that controls the release rate of an active ingredient from the matrix into the skin by forming crystals [22]. Thus, a terahertz pulse wave can penetrate comparatively deeply and provide physical and/or chemical information inside a solid pharmaceutical nondestructively. These advantages suggest that TPI would be applicable as a nondestructive analytical tool not only for process control but also for the quality analysis of commercial products.

In this paper, we compare the terahertz absorption spectra of pure API component with those contained within the solid dosage form. We also obtain terahertz images of four film-coated ciprofloxacin hydrochloride monohydrate (CPFX·HCl·H_2_O) tablets. In this product, the coating has the very important role of protecting the API against degradation caused by light and/or humidity. The authors analyze the coating uniformity and the density of components inside tablets and evaluate the distinguishability among several kinds of commercial tablets that have the same clinical application.

## Experimental

### Materials

To obtain the terahertz absorption spectra of pure materials, CPFX·HCl·H_2_O was purchased from Wako Pure Chemical Industries Ltd. (Osaka, Japan). This compound was used without any further purification. Polyethylene (particle size, <80 μm) used to prepare the sample pellets was purchased from Induchem AG (Volketswil, Switzerland).

CPFX·HCl·H_2_O tablets were obtained from five different commercial sources (Bayer Healthcare Co. Ltd. (Osaka, Japan), Sawai Pharmaceutical Co. Ltd. (Osaka, Japan), Nichi-iko Pharmaceutical Co. Ltd. (Toyama, Japan), Choseido Pharmaceutical Co. Ltd. (Tokushima, Japan) and J-Dolph Pharmaceutical Co. Ltd. (Shiga, Japan)).

All commercial tablets used in this study were a round shape and had a central band. The weight, diameter, and labeled amount were 305 to 310 mg, about 10 mm, and 232.8 mg (as hydrochloride salt monohydrate), respectively.

### Instruments and Measurement Conditions

The terahertz pulsed spectra of the pure CPFX·HCl·H_2_O and the crushed commercial tablets were obtained using TPS Spectra 3000 terahertz spectrometer (TeraView Ltd., Cambridge, UK). Each sample was measured using a spectral range from 120 to 2 cm^−1^ and a spectral resolution of 1.5 cm^−1^. A spectrum was obtained by averaging 1,800 scans and took 1 min. Measurements were obtained by transmittance mode in a dry nitrogen-purged sample compartment. Blackman–Harris term 3 was used as the apodization function. The data were collected using TPS spectra version 1.17.0 (TeraView Ltd.).

Discs were prepared by mixing the pure sample with polyethylene powder at a 10 % (*w*/*w*) concentration, and the two components were mixed well. Then, 400 mg of the mixture was pressed at 2 tons for 2 min to form a disc between 3 and 4 mm thick and with a diameter of 13 mm.

Whole tablets were crushed in a mortar. A portion of the powder equivalent to 10 % API was put into another mortar, up to 200 mg polyethylene powder was added per pellet, and the two were mixed together well. Then a pellet was prepared in the same manner as described above.

Terahertz images of tablets were obtained using the TPI imaga 2000 Coating Scan system (TeraView Ltd.). The operation of this system was well described by Zeitler et al. [11]. Images were acquired in a point-to-point mode with a step size of 100 μm. Three measurements of each tablet were taken, and the measurements together took about 30 min/tablet. Images were analyzed using TPI View version 2.3.10. No sample preparation was required.

## Results and Discussion

### Identification of CPFX·HCl·H_2_O in Tablets Using TPS

**Fig. 1:**
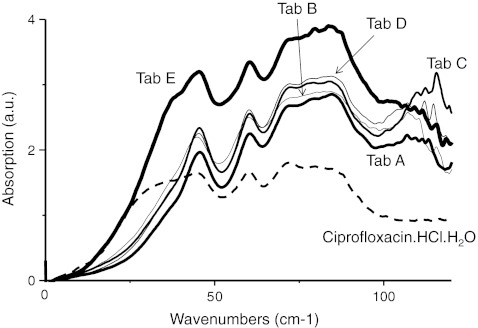
Terahertz spectra of ciprofloxacin hydrochloride monohydrate (*broken line*) and five different commercial tablets (*solid lines*)

The terahertz absorption spectra of the crushed tablets are shown in Fig. 1. Tablets A, B, C, and D show similar spectral features while tablet E exhibits a different spectrum (especially lower wavenumber than 40 cm^−1^). By comparing these spectra to the pure chemical species, we can see that tablets A, B, C, and D are all consistent with each other and with the spectra of CPFX·HCl·H_2_O (broken line). Although the spectral feature of tablet E was different from those of the other tablets, the spectral feature that is lower wavenumber than 40 cm^−1^ was similar to that of CPFX·HCl·H_2_O. According to the enclosed documents for the products, CPFX·HCl·H_2_O is the active ingredient in each product. These results suggest that terahertz spectroscopy can be used to identify API in tablets.

**Fig. 2:**
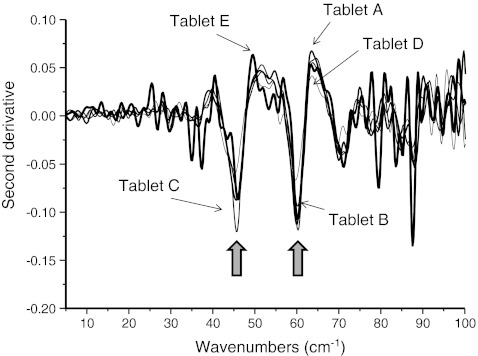
Second derivative terahertz spectra of the five different commercial tablets

Figure 2 shows the second derivative of terahertz absorption spectra obtained from the commercial tablets. The peaks at 60 and 46 cm^−1^ were observed in all of the tablets. The peaks at 88, 85, 84, 79, and 71 cm^−1^ detected in tablet A may be water vapor lines.

Table 1 shows the ingredients listed in the manufacturer’s product literature. This shows that similar ingredients are used for all tablet formulations. Unfortunately, the literature does not disclose the percentage content of each ingredient.

**Table 1:** Ingredients contained in each of five commercial tablets

Tablet A		
Corn starch	Magnesium stearate	Cellulose
Titanium dioxide	Hydroxypropylmethylcellulose (HPMC)	
Carboxymethylstarch soduim	Povidon	Silicate unhydrate
Tablet B		
Corn starch	Cellulose	Magnesium stearate
Titanium dioxide	HPMC	
Macrogol	Crosspovidon	Silicate unhydrate
Tablet C		
Corn starch	Crystallized cellulose	Magnesium stearate
Titanium dioxide	HPMC	
Macrogol 6000	Light anhydrous silicic acid	Tarc
Carboxymethylstarch soduim	Lactose	Carnauba wax
Tablet D		
Corn starch	Hydroxypropylcellulose	Magnesium stearate
Titanium dioxide	HPMC	
Macrogol	Carboxymethylstarch sodium	Citric acid hydrate
Tablet E		
Corn starch	Crystallized cellulose	Magnesium stearate
Titanium dioxide	HPMC 2910	
Macrogol 4000	Crosspovidon	Light anhydrous silicic acid

### Analysis of Quality Attributes of Tablets Using Terahertz Imaging System

#### Density Distribution of Film-Coated Tablets

**Fig. 3:**
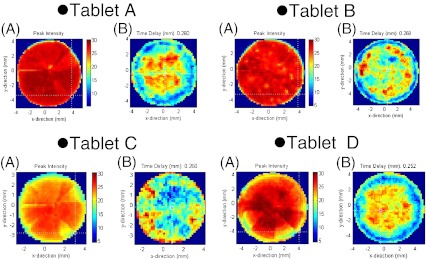
Terahertz images of four different commercial tablets (**a** surface area and **b** at 0.26 mm depth from the surface)

Figure 3 shows the distribution maps of the reflected peak intensities from the surface (A) and 0.26 mm depth (B) of the tablets obtained from each of the measured commercial tablets, respectively. Tablets A and B each have a homogeneous distribution of the peak reflected strength from the surface of the coating, while tablets C and D each have a heterogeneous distribution. As discussed previously, Ho et al. [17] correlated the intensity of reflection to the refractive index of the coating from the equation

$$ R = \frac{{(n - 1)}}{{(n + 1)}} $$

where *R* is the intensity of the reflection and n is the terahertz refractive index of the material. The intensity of reflection from each tablet measured is shown in Fig. 3; these values are labeled with the letter A. They indicate differences between each of the tablets. From the equation described above, we can relate the *R* to the terahertz refractive index of the coating. This is an indication of a change in the density of the coating. During scale-up of a sustained-release coating product, Ho et al. [19] also showed that similar changes in the density of the coating (or in the intensity of reflection from the tablet) can affect product performance. In the case of the tablets studied in this paper, the coating prevents the decomposition of API by light exposure. So, we do not expect the coating to affect the tablets’ dissolution performance. However, this study will provide the sensitivity needed for terahertz measurements against this parameter. We also observe a variation in the intensity of reflection across the surfaces of tablets A and B. This may suggest regions of defective coating or changes in local density on the tablets.

A terahertz dataset allows the experimenter to generate maps at different depths within a tablet without sectioning the tablet. Image B in Fig. 3 shows the distribution of relative refractive indices changing from the tablet surface to a depth of 260 μm. In the images of tablets A and D, the changes in refraction of terahertz pulsed wave by penetrating of component which has different refractive indices are larger at the centers of the tablets than at their outer circles. And tablet B shows comparatively large changes in refraction of terahertz pulsed wave through the wider area of the tablet. In the image obtained from tablet C, small areas having comparatively small changes in refraction of terahertz pulsed wave appear in the center of the tablet. Meanwhile, the edge of the tablet shows larger change in refraction. These observations indicate that features of a tablet’s physical state resulting from the manufacturing process, such as the uneven distribution of granule sizes or the uneven penetration of compression force in a mortar, will change the density of tablet components.

#### In-Depth Terahertz Images

**Fig. 4:**
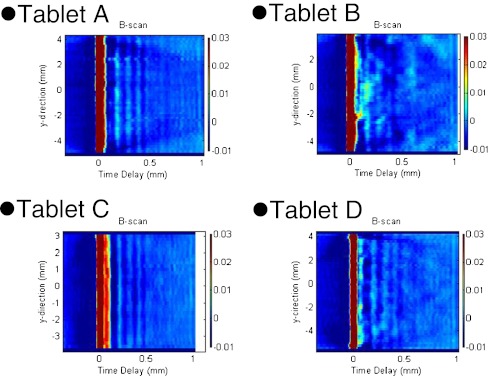
Depth (*B-Scan*) terahertz images of four different commercial tablets (the area to the right of each brown line represents the inside of a tablet)

The depth (B-scan) terahertz images obtained from commercial tablets A–D are shown in Fig. 4. These tablets each have a coating thickness of approximately 100 μm. The left and right sides of the brown line represent air and the inside of the tablet, respectively. The echoes showing several layers formed by compression are observed. Definite layers up to 1 mm depth and up to 0.5 mm depth appear in Tablets A and C, respectively. The indistinct echoes can be seen in Tablet D. On the other hand, indistinct but layer-like echoes are observed in Tablet B. Those observations suggest that unevenly penetrated compression force into the tablet. Further study is necessary to explain the details of these results. However, features of the pre-compression state, such as the particle size distribution of components in a mortar, would be affected by the penetration of compression force in the tablet compaction process. This physical property would be represented as echoes in depth terahertz images. Thus, a depth (B-scan) terahertz image would provide physical information about the effects of the manufacturing process on the tablet’s state and also would sensitively detect changes in manufacturing quality.

#### Distribution of Coating Thickness

**Fig. 5:**
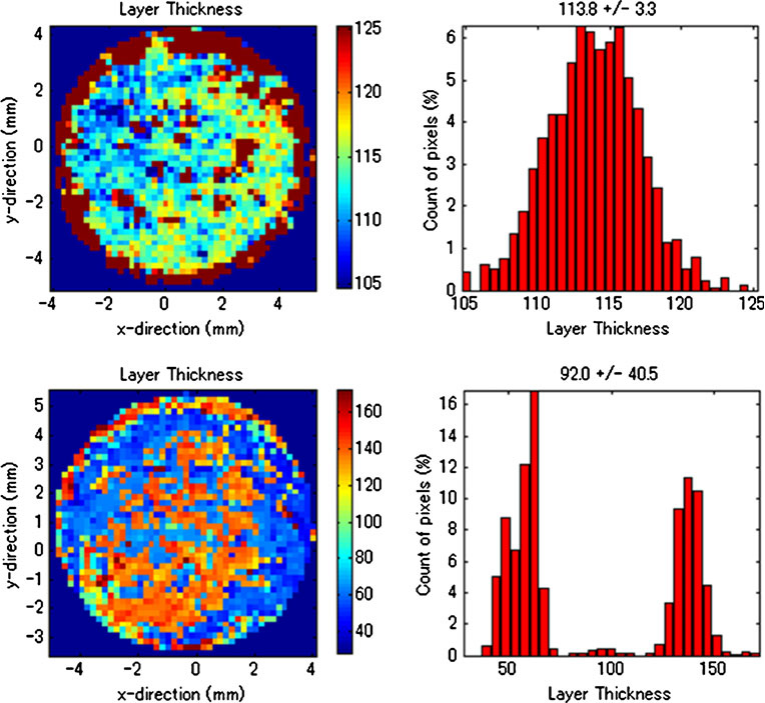
Distribution of coating thickness (*left*) and histograms (*right*) (*upper images*, *Tablet A*; *lower images*, *Tablet D*)

Figure 5 shows the distributions of coating thicknesses obtained from Tablets A and D. A histogram of the coating thickness of each tablet is shown at the right side of this figure. In the case of tablet A, the coating thickness was between 105 and 125 μm, a relatively narrow range of 20 μm. The coating thickness on the outer circuit of each tablet image shows a tendency toward relative thickness, and that on the center shows the opposite tendency. In the case of Tablet D, two peaks in the coating thickness range (40 to 70 and 120 to 150 μm) appear. Moreover, the thin and thick layers are irregularly distributed in the image. This observation definitely indicates that the coating property depends on the coating process. These results suggest that an inappropriate coating process was performed for tablet D.

## Conclusions

A tablet containing relatively large amounts of API (from 75.1 to 82.3 %) would be detected qualitatively by comparison against the characteristic terahertz waveform of API. Terahertz imaging can reveal coating thicknesses and their distributions, the densities of components by compression, and hollows on a tablet surface based on the detection of the delayed reflection of terahertz pulses. Detection of the coating state and changes in the physical state, such as density distribution inside a tablet, would contribute not only to the identification of manufacturing quality but also to qualitative confirmation of commercial tablets including fake (counterfeit) and/or defective products. The TPS and imaging techniques will be useful as nondestructive analytical tools for the quality control of commercial tablets.

## References

[1] Korter TM (2006). Terahertz spectroscopy of solid serine and cysteine. Chem Phys Lett.

[2] Day GM (2006). Understanding the influence of polymorphism on phonon spectra: lattice dynamics calculations and terahertz spectroscopy of carbamazepine. J Phys Chem B.

[3] Allis DG (2006). Solid-state modeling of the terahertz spectrum of the high explosive HMX. J Phys Chem A.

[4] Saito S (2006). Terahertz vibrational modes of crystalline salicylic acid by numerical model using periodic density functional theory. Jpn J Appl Phys Part 1-Regul Pap Brief Commun Rev Pap.

[5] Saito S (2006). Terahertz phonon modes of an intermolecular network of hydrogen bonds in an anhydrous beta-d-glucopyranose crystal. Chem Phys Lett.

[6] Allis DG (2006). Theoretical analysis of the terahertz spectrum of the high explosive PETN. Chem Phys Chem.

[7] Taday PF (2003). Using terahertz pulse spectroscopy to study the crystalline structure of a drug: a case study of the polymorphs of ranitidine hydrochloride. J Pharm Sci.

[8] Walther M (2003). Noncovalent intermolecular forces in polycrystalline and amorphous saccharides in the far infrared. Chem Phys.

[9] Strachan CJ (2004). Using terahertz pulsed spectroscopy to study crystallinity of pharmaceutical materials. Chem Phys Lett.

[10] Zeitler JA (2006). Characterization of temperature induced phase transitions in the five polymorphic forms of sulfathiazole by terahertz pulsed spectroscopy and differential scanning calorimetry. J Pharm Sci.

[11] Zeitler JA (2005). Temperature dependent terahertz pulsed spectroscopy of carbamazepine. Thermochimica Acta.

[12] Strachan CJ (2005). Using terahertz pulsed spectroscopy to quantify pharmaceutical polymorphism and crystallinity. J Pharm Sci.

[13] Zeitler JA (2007). Relaxation and crystallization of amorphous carbamazepine studied by terahertz pulsed spectroscopy. J Pharm Sci.

[14] Kogermann K (2007). Investigating dehydration from compacts using terahertz pulsed, Raman, and near-infrared spectroscopy. Appl Spectrosc.

[15] Zeitler JA (2007). Characterization of drug hydrate systems and dehydration processes using terahertz pulsed spectroscopy. Int J of Pharmaceutics..

[16] Liu H-B (2007). Characterization of anhydrous and hydrated pharmaceutical materials with THz time-domain spectroscopy. J Pharm Sci.

[17] Ho L (2007). Analysis of sustained-release tablet film coats using terahertz pulsed imaging. J Control Release.

[18] Ho L (2008). Applications of terahertz pulsed imaging to sustained-release tablet film coating quality assessment and dissolution performance. J Control Release.

[19] Ho L (2009). Terahertz pulsed imaging as an analytical tool for sustained-release tablet film coating. Eur J Pharm Biopharm.

[20] Fitzgerald AJ (2005). Nondestructive analysis of tablet coating thicknesses using terahertz pulsed imaging. J Pharm Sci.

[21] Zeitler JA (2007). Analysis of coating structures and interfaces in solid oral dosage forms by three dimensional terahertz pulsed imaging. J Pharm Sci.

[22] Sakamoto T (2009). Detection of tulobuterol crystal in transdermal patches using terahertz pulsed spectroscopy and imaging. Pharmazie.

